# Novel Molecular Beacon Probe-Based Real-Time RT-PCR Assay for Diagnosis of Crimean-Congo Hemorrhagic Fever Encountered in India

**DOI:** 10.1155/2014/496219

**Published:** 2014-04-27

**Authors:** Aman Kamboj, Atul Kumar Pateriya, Anamika Mishra, Pradip Ranaware, Diwakar D. Kulkarni, Ashwin Ashok Raut

**Affiliations:** High Security Animal Disease Laboratory, Indian Veterinary Research Institute, Anand Nagar, Bhopal 462022, India

## Abstract

Crimean-Congo hemorrhagic fever (CCHF) is an emerging zoonotic disease in India and requires immediate detection of infection both for preventing further transmission and for controlling the infection. The present study describes development, optimization, and evaluation of a novel molecular beacon-based real-time RT-PCR assay for rapid, sensitive, and specific diagnosis of Crimean-Congo hemorrhagic fever virus (CCHFV). The developed assay was found to be a better alternative to the reported TaqMan assay for routine diagnosis of CCHF.

## 1. Introduction

CCHF is an emerging tick-born zoonosis in India reported for the first time in January 2011 when it killed five people in Gujarat state [1]. CCHFV belongs to genus* Nairovirus* and family Bunyaviridae [2, 3]. It infects a wide range of domestic and wild animals that serve as reservoirs for the virus. The primary route of transmission is either through the bite of a tick vector, mainly* Hyalomma* species, or via direct contact with blood or tissues of viremic animals or humans [3]. To save the patient and to prevent further transmission of the disease, early diagnosis of CCHF is essential. In the present study a novel pair of molecular beacon probe-based real-time RT-PCR (MB rRT-PCR) assay was developed for the sensitive and specific detection of CCHFV in clinical samples. Molecular beacon (MB) is a single-stranded nucleic acid probe comprising three different functional domains: a stem, a loop, and a fluorophore/quencher pair [4]. It is nonfluorescent in the absence of its target sequence but the stem spontaneously opens in the presence of a target to become fluorescent.

## 2. Material and Methods

In the present study MB rRT-PCR assay for rapid, sensitive, and specific detection of CCHF in clinical samples was developed. The present work was performed at the High Security Animal Disease Laboratory, Indian Veterinary Research Institute (IVRI), Bhopal, India, which is a BSL3+ laboratory.

### 2.1. Clinical Samples and RNA Isolation

The clinical samples including blood, serum, and ticks infesting livestock (cattle, buffalo, sheep, and goat) collected from the villages and adjoining areas of earlier reported index cases of CCHF in Gujarat state of India in 2011 were used in the study. The tick samples were processed by triturating the ticks in minimum essential medium (MEM). The triturate was centrifuged at 2,500 rpm for 10 min. Then viral RNA was isolated by using QIAamp Viral RNA Mini-Kit, Qiagen, Germany, following the manufacturer's protocol.

### 2.2. Full-Length Amplification, Cloning, and Sequencing of “S” Segment

The isolated RNA sample was subjected to one-step reverse transcriptase PCR (RT-PCR) using a pair of forward primer, CCHF-SF (5′TCTCAAAGAAACACGTGCCGC3′), and reverse primer, CCHF-SR (5′TCTCAAAGATATCGTTGCCGC3′) [5]. Reaction components used for one-step RT-PCR included 12.5 *μ*L of 2x PCR buffer, 0.5 *μ*L of each forward and reverse primer (10 pm/*μ*L), 1 *μ*L of SS III RT/HF Taq mix (Invitrogen), 5 *μ*L of template RNA, and 5.5 *μ*L of nuclease-free water. The final volume of reaction was 25 *μ*L. One-step RT-PCR was carried out in a thermal cycler (Mastercycler, Eppendorf, USA) using the following cycling condition: cDNA synthesis at 50°C for 30 min; initial denaturation at 94°C for 5 min; 40 cycles of denaturation (94°C, 30 sec); annealing (55°C, 30 sec) and extension (68°C, 30 sec); final extension at 72°C for 10 min; and final hold at 4°C. The full-length amplicons of CCHFV “S” segment were cloned by T/A cloning in pGEM-T Easy vector by using the pGEM-T Easy Vector System I Cloning Kit (Promega, USA) following the manufacturer's protocol. The nucleotide sequencing of the cloned “S” segment was carried out in an automatic DNA Sequencer (Genetic Analyzer ABI 3130, USA) using the BigDye Terminator v. 3.1 Cycle Sequencing Kit (Applied Biosystems, USA). The sequence of the full-length “S” segment was submitted to GenBank online under accession number JX051650.

### 2.3. MB rRT-PCR Optimization

The MB probe and primers based on a sequenced “S” segment (GenBank accession number JX051650) were designed using the OligoArchitect Online v. 3.0 Sigma-Aldrich software from the comparatively conserved region to broaden the diagnostic scope of CCHF and provide an alternative to the test presently available.

The extensive genetic diversity across the CCHFV is challenging for designing the primers and probes for universal diagnosis of CCHFV. The designing of primers and probes for MB rRT-PCR focused primarily on the Asia 2 strains presently circulating in India and its diagnostic applicability for other CCHFV strains bioinformatically assessed. However, in rRT-PCR diagnostics, universal primers and probes for diagnosing different global strains of CCHFV are reported to be designed either by using a consensus sequence [6] or proposing the use of well-defined mixtures of multiple probes [7].

The MB probe and primers were synthesized from Sigma Aldrich (Table 1). The* in vitro* transcribed RNA (IVT-RNA) was produced from a full-length “S” segment pGEM-T clone using a TranscriptAid T7* in vitro* RNA High-Yield Transcription Kit (Thermo Scientific, USA) and used for initial assay optimization.

The MB rRT-PCR was performed in triplicate, in LightCycler 480 Real-Time PCR System (Roche) and optimized using a superscript III one-step RT-PCR Kit (Invitrogen) in a total volume of 12.5 *μ*L containing 1x reaction buffer, 0.5 *μ*L of superscript III RT/platinum Taq mix, 10 pmol of each forward and reverse primer, 2.5 pmol of MB probe, and 1 *μ*L of RNA and with the thermal profile involving cDNA synthesis at 50°C for 30 min, initial denaturation at 95°C for 3 min, followed by 45 cycles of denaturation (95°C, 20 sec), annealing (50°C, 30 sec), and extension (68°C, 20 sec). The fluorescence for each cycle was acquired during the annealing step. No template control (NTC) and no probe control (NPC) were also included in triplicate in each run.

### 2.4. Sensitivity

To determine the lower detection limit, 10-fold serial dilutions (7.6 × 10^9^ to 7.6 copies) of IVT-RNA were made and then a standard curve was plotted.

### 2.5. Specificity

The specificity of the assay was determined using common ruminant viruses, namely, Foot-and-Mouth disease virus, Bovine herpes virus-1, Bovine viral diarrhea 1 and 2, Bovine immunodeficiency virus, and West Nile fever virus.

### 2.6. Reproducibility

The variations in the interassay were assessed by testing the triplicate of each concentration in a single round of real-time PCR and the variations in the intra-assay were assessed by repeating three rounds of MB rRT-PCR. The coefficients of variation (CV) for the cross-point cycle (Cp) values of the intra- and interassay comparisons were determined.

### 2.7. Comparison with TaqMan rRT-PCR

The developed MB rRT-PCR was also compared with the reported TaqMan-based rRT-PCR assay. For TaqMan assay the same protocol was followed as given elsewhere [7]. For determination of sensitivity, specificity, and reproducibility of the TaqMan the same procedure was followed as for MB rRT-PCR assay.

## 3. Results and Discussion

**Figure 1:**
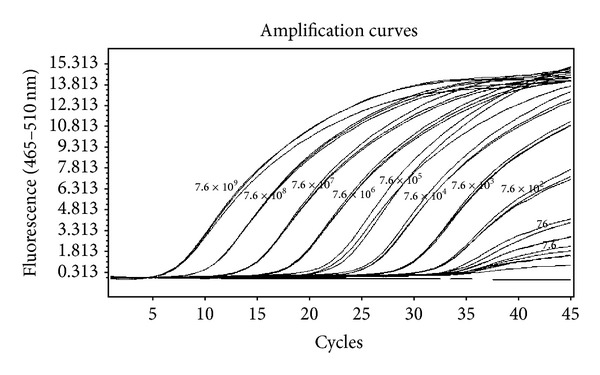
Amplification curves of 10-fold serial dilution of CCHFV IVT-RNA ranging from copy number 7.6 × 10^9^ to 7.6 using MB rRT-PCR. *X*-axis represents the cycle number and *Y*-axis represents the fluorescence acquired at 465–510 nm. The curve shows logarithmic amplification of IVT-RNA dilutions. The numbers on the curves represent the copies of CCHFV IVT-RNA.

**Figure 2:**
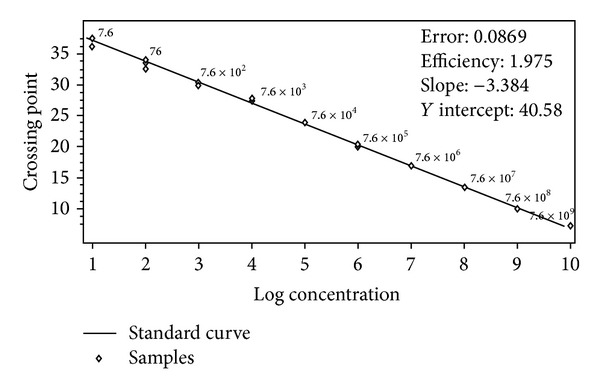
Standard curve for MB rRT-PCR. The assay was linear from 7.6 × 10^9^ to 7.6. *X*-axis represents the IVT-RNA copy number and *Y*-axis represents Cp value.

The MB rRT-PCR protocol yielded a specific sigmoid amplification curve with reproducible results without any background signal (Figure 1). Analytical sensitivity of the assay was determined by plotting a standard curve using 10-fold serial dilutions (7.6 × 10^9^ to 7.6 copies) of* in vitro* transcribed RNA (IVT-RNA). The curve showed −3.384 slope value, 1.975 efficiency, and 0.0869 error rate, which were within the significant range. A sensitivity limit for the assay was 7.6 copies at cut-off Cp 37.04 (Figure 2 and Table 2). High reproducibility of the assay was indicated with 0.21–2.32% and 0.46–3.71% CV of Cp values for intra- and interassay, respectively, which were within the acceptable limit of <5% (Table 2).

The specificity of the assay was determined using common ruminant viruses and no signals were observed with any of the viruses except CCHFV.

The MB rRT-PCR assay was compared with the reported TaqMan rRT-PCR assay [7] using the IVT-RNA standard for analytical sensitivity. We found that MB assay had a comparatively higher efficiency, lower error, and 10-fold higher sensitivity (Table 3, Supplementary Figures 3 and 4in supplementary materials available online at http://dx.doi.org/10.1155/2014/496219).

The developed MB rRT-PCR assay offers a rapid, sensitive, and specific diagnostic alternative for CCHFV infection in clinical samples. For validation of the assay, 212 distinct sera, blood, and tick samples, including positive samples collected from the suspected animals from the Gujarat outbreak area of 2011, were tested (Table 4). The results of the MB assay for these samples were in complete agreement with the results obtained by the conventional nested PCR [8] and the reported TaqMan assay [7].

Molecular diagnostics serve as front-line tools for rapid detection of CCHFV [8]. However, conventional RT-PCR assays are labor intensive, slow, and prone to amplicon contamination. Real-time RT-PCR (rRT-PCR) addresses these problems by allowing rapid, simultaneous amplification, detection, and quantification of target nucleic acids with the use of specific fluorophore-labeled probes or nonspecific DNA-binding dyes. Conventional RT-PCR and rRT-PCR based on SYBR green or a TaqMan probe for detection of CCHFV are widely reported from different parts of the world [6–11]. However, unlike the conventional RT-PCR, MB assay can be performed in a single tube in one step in less time and does not require any postamplification processing of samples that can lead to cross-contamination.

In contrast to the hydrolysis chemistry-based TaqMan assay, during an MB RT-PCR, each molecule of the probe is used several times during the reaction; hence lower concentrations of probes are required in the reaction mix. The probe concentration in the TaqMan assay for CCHF diagnosis was 100 nM [7], while in the MB-RT assay we used probes at a final concentration of 2.5 pmol in the reaction mix. The probe, being one of the expensive components of a diagnostic kit, makes the MB rRT-PCR assay less cost intensive.

## 4. Conclusion

This is the first report on the development of CCHF diagnostic using a molecular beacon platform with sensitivity up to 7.6 copies. The MB rRT-PCR developed is hence proposed as a cost-effective, rapid, specific, and sensitive alternative test for the diagnosis of CCHFV infection.

## Supplementary Material

The developed MB rRT-PCR was also compared with the reported TaqMan-based rRT-PCR assay (Wölfel et al., 2007) [7]. For TaqMan assay the same protocol was followed as given in reported assay. For determination of sensitivity, specificity, and reproducibility of the TaqMan the same procedure was followed as for MB rRT-PCR assay. Figure 3 of supplementary data shows the sigmoid amplification curves of 10-fold serial dilution of CCHFV IVT-RNA ranging from copy no. 7.6 x 10^9^ to 7.6 and figure 4 represents standard curve for TaqMan rRT-PCR. Supplementary data shows MB assay had a comparatively higher efficiency, lower error and 10-fold higher sensitivity than reported TaqMan-based rRT-PCR assay.

## Figures and Tables

**Table 1:** Sequence of primers and MB probe sequence used in developing MB rRT-PCR assay for the diagnosis of CCHF.

Primer/probe	Oligonucleotide sequence (5′-3′)	Nucleotide position (JX051650)^*β*^
MB Probe	6-FAM-CGCGATCATCTCATCTTTGTTGTTCACCTCGATCGCG-BHQ-1	71–93
Forward primer	AGTGTTCTCTTGAGTGCTA	32–50
Reverse primer	CCACAAGTCCATTTCCTT	120–137

6-FAM: 6-carboxyfluorescein.

BHQ-1: Black Hole Quencher-1.

Underlined bases are molecular beacon probe stem.

^*β*^Nucleotide position with reference to NCBI accession number JX051650.

**Table 2:** Intra- and interassay variability for the MB rRT-PCR assay on the basis of Cp values. The limit of detection was determined to be 7.6 copies. The assay was able to generate reproducible results with CV of <5% for both intra- and interassay. The intra-assay variability for TaqMan rRT-PCR assay is also shown on the basis of Cp value. For TaqMan assay the limit of detection is 10-fold lower than MB assay.

CCHFV IVT-RNA copies	MB rRT-PCR				TaqMan rRT-PCR		
	Intra-assay			Interassay	Intra-assay		
	Mean Cp	SD	CV (%)	CV (%)	Mean Cp	SD	CV (%)
7.6 × 10^9^	7.14	0.12	1.68	2.44	9.8	0.35	3.59
7.6 × 10^8^	9.85	0.03	0.30	3.42	13.57	0.49	3.63
7.6 × 10^7^	13.46	0.08	0.59	3.02	18.03	1.08	5.97
7.6 × 10^6^	16.83	0.05	0.30	3.19	22.07	0.93	4.20
7.6 × 10^5^	20.03	0.24	1.20	3.71	25.63	0.25	0.99
7.6 × 10^4^	23.86	0.05	0.21	2.83	29.51	0.43	1.46
7.6 × 10^3^	27.51	0.21	0.76	2.53	31.67	0.29	0.91
7.6 × 10^2^	30.17	0.28	0.93	2.95	34.51	1.64	4.75
7.6 × 10^1^	33.3	0.77	2.31	2.34	32.36	5.85	18.09
7.6	37.04	0.86	2.32	0.46	—	—	—

**Table 3:** Comparison of MB rRT-PCR assay and TaqMan rRT-PCR assay. MB rRT-PCR assay was found to have comparatively higher efficiency, lower error, and 10-fold higher sensitivity than TaqMan rRT-PCR assay.

Assay	Sensitivity	Error	Efficiency
MB rRT-PCR assay	7.6 copies	0.0869	1.975
TaqMan rRT-PCR assay	76 copies	0.162	1.816

**Table 4:** Testing of clinical samples for CCHFV genome using RT-PCR (nested PCR) and TaqMan rRT-PCR for validation of presently developed MB rRT-PCR assay.

Location (districts)	Species	Type of sample	Nested RT-PCR	TaqMan rRT-PCR	MB rRT-PCR	Results
Ahmedabad Mehsana Kutch Dahod Silvassa	Cattle Buffalo Sheep Goat	Serum	03	03	03	Positive
		Blood	03	03	03	
		Ticks	03	03	03	
		Serum	178	178	178	Negative
		Blood	12	12	12	
		Ticks	13	13	13	
Total			**212**	**212**	**212**	** **

## References

[1] Mourya DT, Yadav PD, Shete AM (2012). Detection, isolation and confirmation of Crimean-Congo hemorrhagic fever virus in human, ticks and animals in Ahmadabad, India, 2010-2011. *PLoS Neglected Tropical Diseases*.

[2] Whitehouse CA (2004). Crimean-Congo hemorrhagic fever. *Antiviral Research*.

[3] http://www.who.int/mediacentre/factsheets/fs208/en/.

[4] Tyagi S, Kramer FR (1996). Molecular beacons: probes that fluoresce upon hybridization. *Nature Biotechnology*.

[5] Deyde VM, Khristova ML, Rollin PE, Ksiazek TG, Nichol ST (2006). Crimean-Congo hemorrhagic fever virus genomics and global diversity. *Journal of Virology*.

[6] Garrison AR, Alakbarova S, Kulesh DA (2007). Development of a TaqMan®-minor groove binding protein assay for the detection and quantification of Crimean-Congo hemorrhagic fever virus. *American Journal of Tropical Medicine and Hygiene*.

[7] Wölfel R, Paweska JT, Petersen N (2007). Virus detection and monitoring of viral load in Crimean-Congo hemorrhagic fever virus patients. *Emerging Infectious Diseases*.

[8] Drosten C, Kümmerer BM, Schmitz H, Günther S (2003). Molecular diagnostics of viral hemorrhagic fevers. *Antiviral Research*.

[9] Drosten C, Göttig S, Schilling S (2002). Rapid detection and quantification of RNA of Ebola and Marburg viruses, Lassa virus, Crimean-Congo hemorrhagic fever virus, Rift Valley fever virus, dengue virus, and yellow fever virus by real-time reverse transcription-PCR. *Journal of Clinical Microbiology*.

[10] Duh D, Saksida A, Petrovec M, Dedushaj I, Avsic-Zupanc T (2006). Novel one-step real-time RT-PCR assay for rapid and specific diagnosis of Crimean-Congo hemorrhagic fever encountered in the Balkans. *Journal of Virological Methods*.

[11] Kalvatchev N, Christova I (2008). One step RT-PCR for rapid detection of Crimean-Congo haemorrhagic fever virus. *Biotechnology and Biotechnological Equipment*.

