# Supplementary material for: Novel Molecular Beacon Probe-Based Real-Time RT-PCR Assay for Diagnosis of Crimean-Congo Hemorrhagic Fever Encountered in India
Source: Biomed Res Int. 2014 Apr 27;2014:496219. doi: 10.1155/2014/496219 (PMC4022296; doi:10.1155/2014/496219)
Supplement: Supplementary file 1 — The developed MB rRT-PCR was also compared with the reported TaqMan-based rRT-PCR assay (Wölfel et al., 2007) [7]. For TaqMan assay the same protocol was followed as given in reported assay. For determination of sensitivity, specificity, and reproducibility of the TaqMan the same procedure was followed as for MB rRT-PCR assay. Figure 3 of supplementary data shows the sigmoid amplification curves of 10-fold serial dilution of CCHFV IVT-RNA ranging from copy no. 7.6 x 10^9^ to 7.6 and figure 4 represents standard curve for TaqMan rRT-PCR. Supplementary data shows MB assay had a comparatively higher efficiency, lower error and 10-fold higher sensitivity than reported TaqMan-based rRT-PCR assay. [file 496219.f1.pdf]

Supplementary material

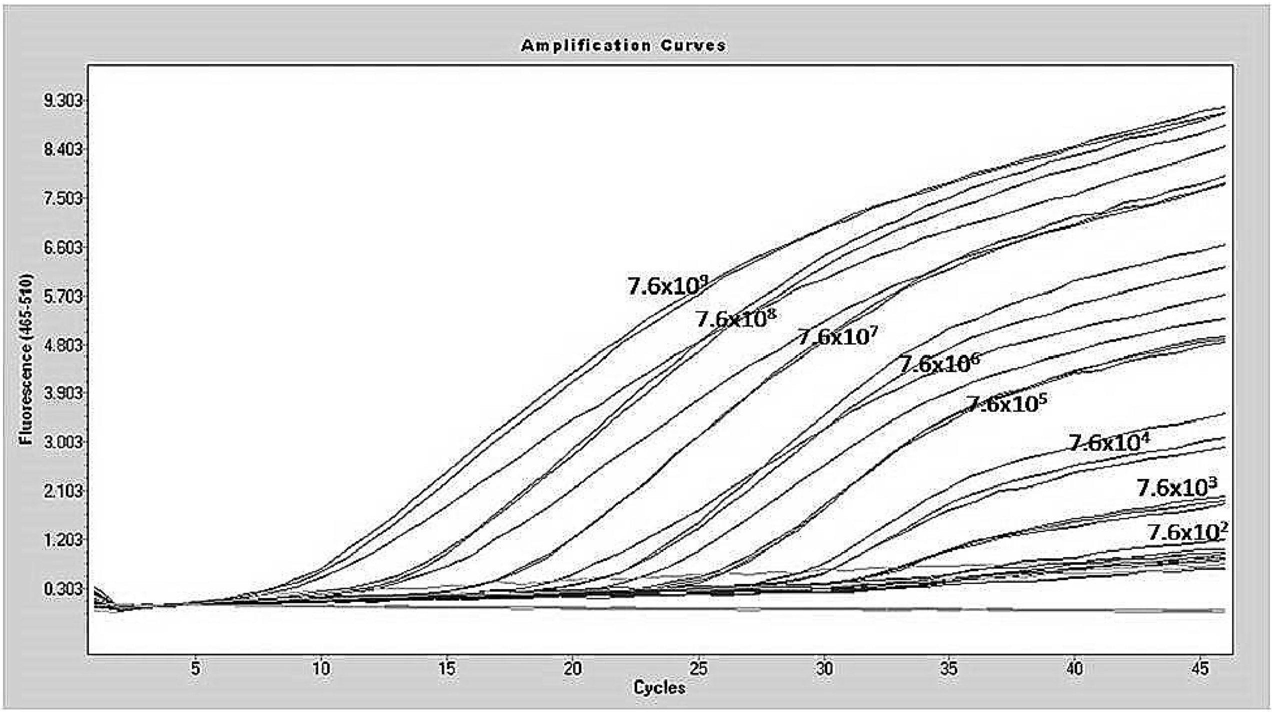

FIGURE 3:Amplification curves of 10-fold serial dilution of CCHFV IVT-RNA ranging from copy no. 7.6 x 10^9 to 7.6 using TaqMan rRT-PCR. X-axis represents the cycle no. and Y-axis represents the fluorescence acquired at 465-510 nm. The curve shows logarithmic amplification of IVT-RNA dilutions. The numbers on the curves represents the copies of CCHFV IVT-RNA.

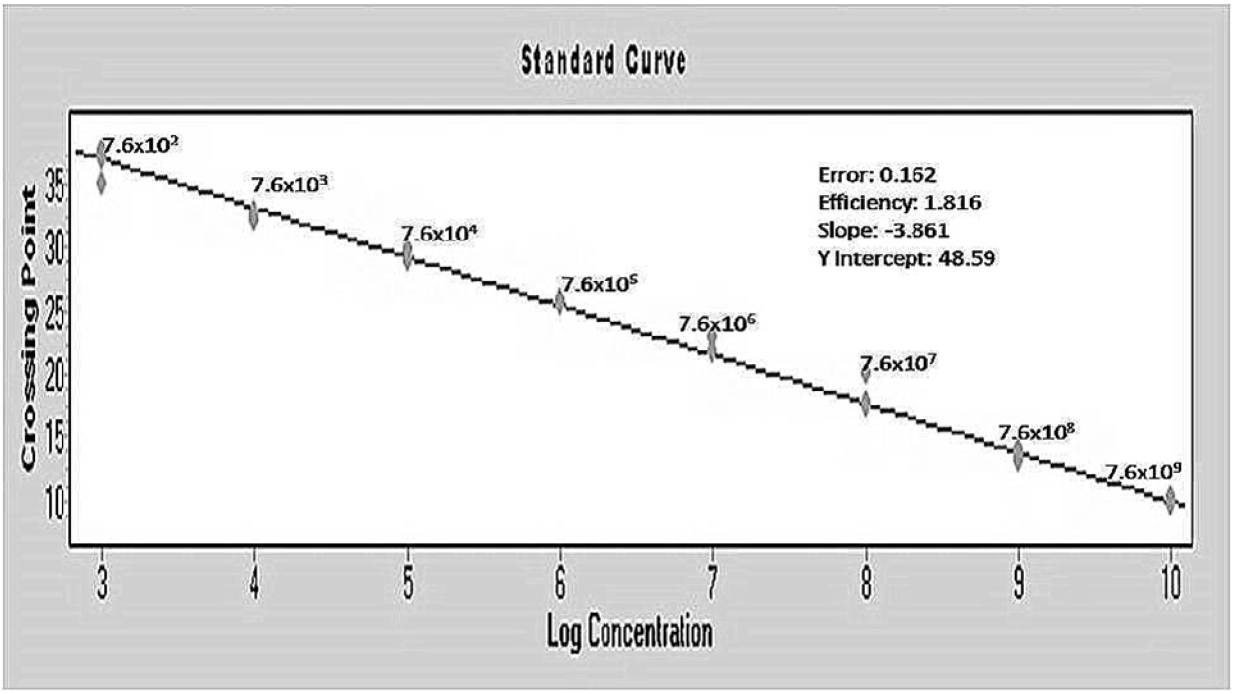

FIGURE 4: Standard curve for TaqMan rRT-PCR. The assay was linear from $7.6x10^9$ to 7.6 x 10^2. X-axis represents the IVT-RNA copy number and Y-axis represents Cp value.
